# The National Conference on Health Disparities Student Research Forum

**DOI:** 10.1007/s13187-021-02082-3

**Published:** 2021-10-16

**Authors:** Marvella E. Ford, Angela M. Malek, Erica Martino, Latecia Abraham-Hilaire, Oluwole Ariyo, Dana Burshell, Gloria Callwood, Laura Campbell, Kimberly Cannady, Courtney Chavis, Brittney Crawford, Andie Edwards, Victoria Findlay, Rita Finley, Chamiere Greenaway, Tonya Hazelton, Monique Hill, Marion Howard, Kendrea D. Knight, Vanessa Lopez-Littleton, Lloyd Moore, Diandra Randle, David E. Rivers, Judith D. Salley, Terry Seabrook, Sabra Slaughter, James B. Stukes, Roland J. Thorpe, LaVerne Ragster

**Affiliations:** 1 Department of Public Health Sciences, Medical University of South Carolina, Charleston, SC 29425 USA; 2 Hollings Cancer Center, Medical University of South Carolina, Charleston, SC 29425 USA; 3 Department of Public Information & Community Outreach Main Library, Medical University of South Carolina, Charleston, SC 29425 USA; 4 Department of Biology, Allen University, Columbia, SC 29204 USA; 5 Carolina Population Center, University of North Carolina at Chapel Hill, Chapel Hill, NC 27516 USA; 6 School of Nursing, University of the Virgin Islands, Charlotte Amalie, 00802 US Virgin Islands; 7 College of Medicine, Medical University of South Carolina, Charleston, SC 29425 USA; 8 Department of Academic Affairs Faculty, Medical University of South Carolina, Charleston, SC 29425 USA; 9 Department of Pathology and Laboratory Medicine, Medical University of South Carolina, Charleston, SC 29425 USA; 10 Department of Pathology and Anatomy, Morehouse School of Medicine, Atlanta, GA 30310 USA; 11 Public Policy Department, Amerihealth Caritas Family of Companies, Philadelphia, PA 19113 USA; 12 College of Nursing, Medical University of South Carolina, Charleston, SC 29425 USA; 13 California State University Monterey Bay, 100 Campus Center, Seaside, CA 93955 USA; 14 National Environmental Justice Conference and Moore Companies, Washington, D.C 20006 USA; 15 Department of Biological & Physical Sciences, South Carolina State University, Orangeburg, SC 29115 USA; 16 The Space Company, 82 ½ Spring Street, Charleston, SC 29403 USA; 17 Department of Medicine, Center for Health Disparities Research, Medical University of South Carolina, Charleston, SC 29425 USA; 18 Program for Research On Men’s Health, Hopkins Center for Health Disparities Solutions, Johns Hopkins University Bloomberg School of Public Health, Baltimore, MD 21205 USA; 19 University of the Virgin Islands, Charlotte Amalie, 00802 US Virgin Islands

**Keywords:** United States, Health disparities, Underrepresented students, Conference, Professional development

## Abstract

The annual National Conference on Health Disparities (NCHD) was launched in 2000. It unites health professionals, researchers, community leaders, and government officials, and is a catalyzing force in developing policies, research interventions, and programs that address prevention, social determinants, health disparities, and health equity. The NCHD Student Research Forum (SRF) was established in 2011 at the Medical University of South Carolina to build high-quality biomedical research presentation capacity in primarily underrepresented undergraduate and graduate/professional students. This paper describes the unique research training and professional development aspects of the NCHD SRF. These include guidance in abstract development, a webinar on presentation techniques and methods, a vibrant student-centric conference, and professional development workshops on finding a mentor and locating scholarship/fellowship funding, networking, and strategies for handling ethical issues in research with mentors. Between 2011 and 2018, 400 undergraduate and graduate/professional students participated in the NCHD SRF. Most students were women (80.5%). Approximately half were African American or black (52.3%), 18.0% were white, and 21.3% were of Hispanic/Latinx ethnicity. The NCHD SRF is unique in several ways. First, it provides detailed instructions on developing a scientific abstract, including content area examples. Second, it establishes a mandatory pre-conference training webinar demonstrating how to prepare a scientific poster. Third, it works with the research mentors, faculty advisors, department chairs, and deans to help identify potential sources of travel funding for students with accepted abstracts. These features make the NCHD SRF different from many other conferences focused on students’ scientific presentations.

**Supplementary Information:**

The online version contains supplementary material available at 10.1007/s13187-021-02082-3.

## Introduction

### Statement of the Problem

#### The Shortage of Diverse Biomedical Scientists in South Carolina and the United States

The Millennial Generation, comprising 26% of the population, is the most diverse in United States (US) history. However, young adults are not entering biomedical science careers (science, technology, engineering, and mathematics, and specifically physical, health, and life sciences [STEM]) at commensurate rates as previous generations, especially those who are racially and ethnically diverse, from rural areas, and from low socioeconomic-position backgrounds [3] Since 2000, the percentage of underrepresented minorities receiving degrees in engineering and the physical sciences has been flat [3]. According to a 2018 PEW Research Center report, African American and Hispanic workers continue to be underrepresented in the STEM workforce. African Americans comprise 11% of the US workforce overall, but represent only 9% of STEM workers, while Hispanics comprise 16% of the US workforce but represent only 7% of all STEM workers [7]. However, within job clusters, the representation of African Americans and Hispanics varies widely. To cite an example, 37% of licensed practical and vocational nurses are either African American or Hispanic.

In contrast, healthcare jobs requiring higher levels of education and with higher pay scales have smaller percentages of workers who are African American or Hispanic, including physicians and surgeons (11%), pharmacists (10%), dentists (9%), and physical therapists (9%). Only 5% of optometrists, veterinarians, and chiropractors are African American or Hispanic [7]. At the same time, the prevalence of chronic diseases (including cancer, diabetes, stroke, heart disease, obesity, and oral diseases) in the US is rapidly increasing, to the extent that nearly half (45% of the 329,987,196 people in the US) suffer from at least one of these diseases [11]. Chronic diseases, by definition, last for more than 1 year and are often accompanied by minor to significant functional limitations [4, 5]. Therefore, the demand for chronic disease prevention, screening, and treatment services will grow commensurately over the next two decades as the proportion of older adults in the US increases. It is imperative that we equip larger numbers of diverse, post-Millennial Generation students with the skill sets, and cultivate in them the motivation, required to attain and excel in biomedical careers as part of a solution to the problem of insufficient healthcare workers to care for an aging America.

To improve health outcomes, investigators must understand the science behind the disease. Chronic disease is a multi-step disease process resulting from a series of biologic, social, and environmental changes that abrogate normal cellular growth controls [6]. Advances in chronic disease research will require well-trained, interactive, and productive scientists with the ability to understand and manipulate molecular, cellular, and genetic events within a physiological context, as well as social and environmental contexts. A landmark review by Ginther et al*.* [8] noted that African Americans are 10% less likely than whites to receive National Institutes of Health (NIH) Research Project Grant (R01) funding, a marker of independent investigator status, even after controlling for demographic characteristics, education and training, and research productivity among other measures.

Given the potential for dramatic workforce shortages due to the reasons mentioned above, particularly among underrepresented populations, it is imperative to devise strategies and execute well-planned training programs designed to enhance the scope and diversity of the next generation of chronic disease researchers and clinician scientists. As noted by the Institute of Medicine [10], greater diversity among medical researchers and clinicians leads to improved access to care among racially and ethnically diverse populations, greater patient choice and satisfaction, improved patient-provider communication, and better educational experiences for biomedical students during their training [1, 2, 9]. Increasing the number of diverse investigators who are well-trained in rigorous methodological and analytical principles of research is a critical step toward increasing capacity in health equity research and toward decreasing disparities in health outcomes.

#### Rationale for the National Conference on Health Disparities Student Research Forum

The central purpose of the National Conference on Health Disparities (NCHD) Student Research Forum (SRF) is to build capacity in biomedical sciences research in primarily underrepresented undergraduate and graduate/professional students. To address the shortage of racially/ethnically diverse biomedical professionals in the US workforce, Congressman Jim Clyburn and former Congresswoman Donna Christensen, who co-lead the annual NCHD, decided to include a SRF as part of the NCHD. A critical component of the NCHD is the SRF, which was developed in 2011 at the Medical University of South Carolina (MUSC) to build high-quality research presentation capacity in underrepresented undergraduate and graduate/professional students across the US.

## Methods

To maximize the benefits of the 1-day annual NCHD SRF, a strategic approach to training and mentoring undergraduate and graduate/professional students in the art of scientific presentations is included. The methods used in developing and implementing the SRF are described below.

### Call for Abstracts

The NCHD SRF Planning Committee issues a national Call for Abstracts four months prior to the due date of the abstracts. In keeping with the theme of training the next generation of health disparities researchers and policy makers, the Call for Abstracts includes the statement that the mission of the NCHD is to focus on policies, research interventions, and programs that address prevention, social determinants, and personal responsibility in reducing health disparities and promoting health equity. Abstracts from the following categories are invited for submission: (a) basic sciences, (b) clinical sciences, (c) population sciences/behavioral sciences/social sciences, and (d) environmental sciences. The Call for Abstracts also notes that required national poster and oral presentation training webinars will take place prior to the NCHD SRF for students whose abstracts were accepted, to enhance the students’ presentation skills.

### Abstract Submission Guidelines

The guidelines for abstract submissions are posted on the NCHD website. The guidelines document explicitly states that all abstracts must include the following information: (1) hypothesis or statement about the problem being investigated and why the research is important, (2) methods, (3) results and discussion of findings, (4) conclusions and future research directions, and (5) acknowledgement of funder(s).

The guidelines for abstract submissions document also includes information to assist the students as they prepare their abstracts. This information is more explicit than the information that would typically be included in a call for abstracts to serve as a learning tool for the students: (1) Only one poster abstract can be submitted per student as primary author. However, a student may be listed as a co‐author on a second abstract. (2) Students working in the same laboratory must independently submit original abstracts. Identical abstracts submitted by different students will be automatically rejected. (3) Approval must be obtained from all co‐authors listed on the abstract; failure to do so will result in the immediate rejection of the abstract. (4) Students must obtain approval from their faculty advisor(s)/research mentor(s) before submitting the abstract; failure to do so will result in the immediate rejection of the abstract. (5) Abstracts must be written by the student and reviewed by their faculty advisor or research mentor. (6) Abstracts must adhere to the highest quality standards—with correct grammar, spelling, and sentence structure (i.e., with editing and proofreading prior to submission). (7) Data tables and figures are not to be included with the abstract. (8) Abstracts are limited to a maximum of 300 words.

The guidelines for abstract submissions document also include links to the following helpful guides on developing an abstract:

- http://writingcenter.unc.edu/handouts/abstracts/
- http://rc.rcjournal.com/content/49/10/1206.full.pdf
- http://www.chemistryviews.org/details/education/2709521/Tips_for_Writing_Better_Science_Papers_Abstract_3.html

### Incentivizing Students from Academic Institutions Across the US to Submit Abstracts

To further incentivize the students to submit their abstracts for the NCHD SRF, the Call for Abstracts also notes that tiered scholarships to support hotel costs will be available on a limited basis for students with the highest-ranked abstracts, based on the abstract review scores. The students may apply for the competitive scholarships for hotel support when they submit their abstracts. The Call for Abstracts also includes a statement that cash prizes will be awarded to the 1st, 2nd, and 3rd place ranked poster presentations in the undergraduate and graduate/professional student categories, respectively ($500, $300, and $200). Furthermore, the two oral presenters each receive a cash award of $100 as well as a scholarship to cover the costs of their airfare and hotel charges. The funds for the airfare, hotel costs, poster presentation cash prizes, and the oral presentation cash awards are awarded by the NCHD.

The Wufoo online form builder platform is used for the abstract submission process. This platform allows for the collection of supplemental data related to the abstract submission, such as the student’s contact information (name, mailing address, email address, telephone number); gender; undergraduate or graduate/professional student status; college or university; name and email address of the student’s dean, research mentor, faculty advisor, and department chair; category of research (i.e., clinical sciences); abstract title and authors; the actual abstract; whether a student was applying for a travel scholarship; and the level of travel scholarship for which the student is applying (hotel costs only). The students are responsible for paying the conference registration fees, which include breakfast, lunch, and evening networking receptions.

### Responding to Students’ Questions During the Abstract Submission Process

During the abstract submission process, students send the NCHD SRF organizers several different questions. Examples of the questions and responses are listed below.

- Question: Am I allowed to present a poster on work that I completed during a recent undergraduate internship if I am now in graduate/professional school? Response: Yes. Your abstract will still be judged in the graduate/professional student category.
- Question: May I see my abstract review comments? Response: Yes, we will send the comments to you.
- Question: Several of my classmates and I worked on the same project. Can we each submit an abstract about the project? Response: Yes, as long as each abstract focuses on a different analytic outcome related to the project.
- Question: I work in a research team. Other students on my team submitted abstracts that were accepted but mine was not. Could you please explain this? Response: Each abstract is reviewed independently by a set of scientific reviewers based on the listed review criteria.
- Question: Could I choose to give an oral vs poster presentation? Response: The abstracts with the highest scores in the undergraduate and the graduate/professional student category will be selected for oral presentation in addition to poster presentation.

### Abstract Review Process

Abstracts are evaluated by faculty from across the US who serve on the NCHD SRF Planning Committee and/or the Abstract Review Sub-Committee. Reviewers are assigned abstracts to review in either the undergraduate or the graduate/professional student categories, resulting in a separate set of reviewers for each category. Each abstract is assigned to two reviewers who independently evaluate the abstract using the following five criteria, each of which has a maximum value of 20 points, for a total of 100 possible points: (1) originality and innovation, (2) scientific content supported by quantitative information, (3) merit of the research, (4) quality of the written content, and (5) relevance and adherence to guidelines and format. Abstracts ranked with a score that is equivalent to or exceeds the mean score for the undergraduate or the graduate/professional student categories, respectively, are accepted for presentation. Abstract acceptance/non-acceptance notifications as well as selection for the travel awards are sent to the students after the abstract reviews have been completed. The notifications are sent at least 2 months prior to the conference to allow the students time to prepare their poster and/or oral presentations, and to identify alternative and/or supplemental sources of travel funds.

Additionally, unlike most other student conferences, a Financial Assistance Sub-Committee of the SRF, with the student’s permission, contacts the research mentors, faculty advisors, department chairs, and deans of each student whose abstract was accepted, to inform them that the student’s abstract was accepted and to work with them to identify potential sources of funding to support the student’s participation in the NCHD SRF. The Financial Assistance Sub-Committee members first contacts each student’s research mentor and faculty advisor. If those contacts do not lead to the successful identification of funding sources, then the sub-committee members contact the student’s department chair and/or dean.

### National Webinar Training in Poster and Oral Presentations

To assist the students whose abstracts were accepted with preparing their posters and oral presentations, the NCHD SRF conducts a national training webinar which was initiated in 2014 to improve the quality of the scientific research that is presented. The webinar includes key points related to developing an effective poster presentation, including the appropriate design and layout, the order of the information that is presented (e.g., Introduction, Purpose, Sample, Methods, Results, Discussion, Conclusions, and References), the identification of the audience for the poster presentation, the proper labeling of figures and graphs in the poster presentation, and using a software program to develop the presentation. Additionally, the dimensions of the poster boards that will be used are sent to the students along with the required poster dimensions, which are dependent on the size of the poster boards that are rented at the conference location each year.

### NCHD SRF Agenda

The NCHD SRF takes place from 7:30 a.m. to 3 p.m. on a Wednesday in late June, and the day prior to the NCHD conference which occurs from Thursday to Saturday. The NCHD SRF agenda includes: (1) a welcome and overview; (2) oral presentations by the undergraduate and graduate/professional students, respectively, with the highest abstract scores; (3) poster presentations judged by faculty from across the US during the conference; (4) a keynote speaker presentation during lunch, followed by a breakout for undergraduate and graduate/professional students; (5) roundtable discussions held for graduate/professional students in the areas of mentoring/networking, professional ethics, and identifying research opportunities; (6) roundtable discussions held for undergraduate students about applying to graduate or professional school; (7) a workshop held for undergraduate students on preparing abstracts for future scientific meetings; and (8) a presentation of an interactive learning module by a National Library of Medicine staff member.

### Poster Review Criteria

The NCHD SRF includes three main categories by which the posters are evaluated, as well an overall review category. The four review categories are: research content (42 points maximum), presentation delivery (30 points maximum), poster quality (24 points maximum), and overall presentation quality (6 points maximum). Therefore, the maximum score for each poster is 102 points.

The research content category includes seven components: (1) the research has merit and makes a contribution to the field of study, (2) the abstract accurately summarizes the research presented, (3) the introduction provides brief yet pertinent information about the project, (4) the methodology is appropriate for the research presented, (5) appropriate methods of analysis for interpretation are utilized, (6) the results are detailed and explained accurately, and (7) the presentation provides implications for future research and/or practical application. A maximum score of 6 points is possible for each component for which the scale ranges from 1 (Needs Improvement) to 6 (Above Average).

The presentation delivery category includes five components: (1) the presenter responded fluently to questions regarding their work, (2) the presenter was enthusiastic about the research and utilizes both the poster and personal knowledge of the subject to present the research, (3) the presenter had a strong understanding of the information and the topic, (4) the presenter was an active participant in presenting the research (interested in questions, outgoing, knowledgeable about the research topic), and (5) the presenter’s mannerisms (eye contact and gestures) contributed to the explanation of the research. A maximum score of 6 points is possible for each component for which the scale ranges from 1 (Needs Improvement) to 6 (Above Average).

The poster quality category includes four components with a maximum score of 6 points each: (1) the poster content was grammatically correct, (2) the presenter used the available poster space effectively, (3) all required elements (abstract, introduction, procedures, discussion, etc*.*) were apparent and easy to follow, and (4) all figures, charts, images, and graphs were properly presented and contained information relevant to the central topic. A maximum score of 6 points is possible for each component for which the scale ranges from 1 (Needs Improvement) to 6 (Above Average). The final category by which the posters were judged is an overall category. It provides a scale ranging from 1 (Needs Improvement) to 6 (Above Average) and reflects the judges’ overall evaluative review of the poster.

## Results

Between 2011 and 2018, a total of 407 undergraduate and graduate/professional students participated in the NCHD SRF or 58 students per year on average, as shown in Table 1. Most participants were female (80.8%). With regard to race and ethnicity, approximately half of the NCHD SRF students were African American or black (52.1%), 17.9% were white, 21.6% were of Hispanic/Latinx ethnicity, and the remainder (8.4%) were of Asian or other racial/ethnic groups including American Indian or Alaska Native, Native Hawaiian or Other Pacific Islander, and unknown race. Table 1 describes the gender characteristics of all of the student participants. However, due to the small number of American Indian/Alaska Native, Native Hawaiian or Other Pacific Islander, and Asian student participants, Table 1 only describes the racial/ethnic characteristics for African American or black, white, and Hispanic/Latinx student participants.

**Table 1:** Demographic characteristics of the National Conference on Health Disparities (NCHD) Student Research Forum (SRF) participants by year and location^a^

	2011 Charleston, SC, *n* = 57 (%)	2012 Little Rock, AR, *n* = 64 (%)	2013 St. Thomas, US Virgin Islands, *n* = 87 (%)	2014 Long Beach, CA, *n* = 64 (%)	2016 Washington, DC, *n* = 61 (%)	2017 New Orleans, LA, *n* = 36 (%)	2018 Philadelphia, PA, *n* = 38 (%)	*p-value*	Total, *n* = 407 (%)
Gender									
Male	12 (21.1)	13 (20.3)	12 (13.8)	9 (14.1)	13 (21.3)	7 (19.4)	12 (31.6)	0.29	78 (19.2)
Female	45 (78.9)	51 (79.7)	75 (86.2)	55 (85.9)	48 (78.7)	29 (80.6)	26 (72.2)		329 (80.8)
Race/Ethnicity^b^									
African American or Black	39 (68.4)	35 (61.4)	63 (77.8)	15 (24.6)	28 (50.9)	10 (35.7)	22 (64.7)	0.00	212 (56.8)
White	7 (12.3)	8 (14.0)	6 (7.4)	11 (18.0)	18 (32.7)	12 (42.9)	11 (32.4)		73 (19.6)
Hispanic/Latinx	11 (19.3)	14 (24.6)	12 (14.8)	35 (54.7)	9 (16.4)	6 (21.4)	1 (2.9)		88 (23.6)

*Abbreviations*: *SC* South Carolina, *AR* Arkansas, *US* United States, *CA* California, *DC* District of Columbia, *LA* Louisiana, *PA* Pennsylvania, *SD* standard deviation

^a^The NCHD SRF was not held in 2015

^b^Participants of Asian (*n* = 27) and other racial/ethnic groups (*n* = 7; includes American Indian or Alaska Native, Native Hawaiian or Other Pacific Islander, and unknown race) are not shown

**Fig. 1:**
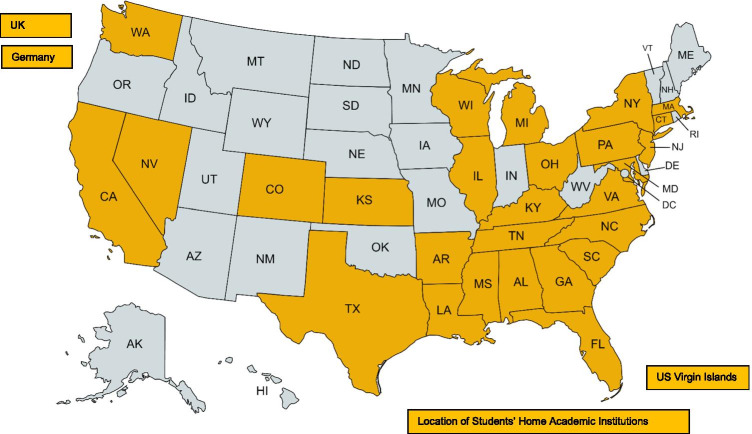
National and international locations of academic institutions of students participating in the National Conference on Health Disparities (NCHD) Student Research Forum (SRF), 2011-2018. Note: The NCHD SRF was not held in 2015

Figure 1 shows that the NCHD SRF participants represent the East Coast, West Coast, Southeast, and Midwest regions of the US, as well as international locations. The Supplemental Table (Online Resource 1) displays the specific location of each NCHD and the academic institutions of SRF participants by year. In addition, the categories of presented abstracts and poster winners of the NCHD SRF for 2011–2018 are displayed by year in Table 2.

**Table 2:** Categories of presented abstracts and poster winners of the National Conference on Health Disparities (NCHD) Student Research Forum (SRF), 2011–2018*

$\underline{2011\ (n=21)}$ Undergraduate students Basic Sciences: 4 Clinical Sciences: 3 Population Sciences/Behavioral Sciences/Social Sciences: 3 Environmental Sciences: 0 Graduate/professional students Basic Sciences: 1 Clinical Sciences: 0 Population Sciences/Behavioral Sciences/Social Sciences: 10 Environmental Sciences: 0 Undergraduate student poster winners 1st place: N/A 2nd place: N/A 3rd place: N/A Graduate/professional student poster winners 1st place: N/A 2nd place: N/A 3rd place: N/A	$\underline{2012\ (n=39)}$ Undergraduate students Basic Sciences: 2 Clinical Sciences: 2 Population Sciences/Behavioral Sciences/Social Sciences: 11 Environmental Sciences: 0 Graduate/professional students Basic Sciences: 3 Clinical Sciences: 2 Population Sciences/Behavioral Sciences/Social Sciences: 19 Environmental Sciences: 0 Undergraduate student poster winners 1st place: Madhulika Vulimiri, University of North Carolina at Chapel Hill, Poster Title: Patient and Provider Perspectives of Community-based Diabetes Health Promoter-led Intervention 2nd place: Kai Carey, Philander Smith College, Poster Title: Role of TGM2 and CIN85 in the Radiation Resistance of Pancreatic Cancer Cells 3rd place: Lakeisha Watson, University of North Carolina at Chapel Hill, Poster Title: Risk factors and HIV Education in Rural Counties in North Carolina among African Americans Graduate/professional student poster winners 1st place: Brittney Mull, California State University- Long Beach, Poster Title: Discrimination in Health Care Settings: Perspectives of Latino Patients 2nd place: Lizette Alvarez, California State University- Long Beach, Poster Title: Previous Nutrition Education and its Influence on the Outcomes of Nutrition Education Programs at Point of Purchase 3rd place: Feana Francis Devaraj, University of Arkansas for Medical Sciences, Poster Title: Naturally Derived Compounds and their Cytotoxicity in Pancreatic Cancer Cell Lines
$\underline{2013\ (n=32)}$ Undergraduate students Basic Sciences: 0 Clinical Sciences: 1 Population Sciences/Behavioral Sciences/Social Sciences: 5 Environmental Sciences: 1 Graduate/professional students Basic Sciences: 1 Clinical Sciences: 2 Population Sciences/Behavioral Sciences/Social Sciences: 22 Environmental Sciences: 0 Undergraduate student poster winners 1^st^ place: Kendra Hearn, Spellman College, Poster Title: Lapatinib and Neratinib: Using Combination Drug Therapy to Advance the Effect of the Treatment of HER2 Positive Breast 2^nd^ place: Nicole Crawford, Georgia Southern University, Poster Title: Intimate Partner Violence: A Silent Phenomenon 3^rd^ place: Sarah Wolf, University of the Virgin Islands, Poster Title: Factors Contributing to Fecal Indicator Bacteria in Brewer’s Bay, USVI Graduate/professional student poster winners 1^st^ place: Angelika Clarke, California State University- Long Beach, Poster Title: Risk Factors for Early Pubertal Onset in African American Girls 2^nd^ place: Yamisha Rutherford, Morehouse School of Medicine, Poster Title: Weight Change as a Predictor of Physical Health Among African American Breast Cancer Survivors 3^rd^ place: Eugenia Maravilla, California State University-Long Beach, Poster Title: The Potential Antimicrobial Activity of Locusta Migratoria Apolipophorin III	$\underline{2014\ (n=55)}$ Undergraduate students Basic Sciences: 2 Clinical Sciences: 1 Population Sciences/Behavioral Sciences/Social Sciences: 15 Environmental Sciences: 0 Graduate/professional students Basic Sciences: 1 Clinical Sciences: 0 Population Sciences/Behavioral Sciences/Social Sciences: 34 Environmental Sciences: 2 Undergraduate student poster winners 1^st^ place: Nanci Alanis Alcantara, University of Illinois at Chicago, Poster Title: How do Different Stressors Relate to Body Mass Index Among U.S. and Immigrant-born Latinas? 2^nd^ place: Joaquin Magana, Occidental College, Poster Title: Developing a qPCR Assay to Quantify Relative Telomere Length; a Tool to Investigate the Basic Science of Health Disparities 3^rd^ place: Marina Armendariz, California State University- Long Beach, Poster Title: Obesity- related Eating Habits Heighten Risk for Type 2 Diabetes among at-risk Latino College Students Graduate/professional student poster winners 1^st^ place: Tanya Troy, Wayne State University School of Medicine, Poster Title: Advance Directives in an Older African American Population: What are the Attitudes and Barriers to Completion? A Pilot Study 2^nd^ place: Jasmine Travers, Columbia University School of Nursing, Poster Title: Does State Legislation Improve Nursing Workforce Diversity? 3^rd^ place: Lorena Rodriguez, California State University- Long Beach, Poster Title: Does Nutritional Training Increase Knowledge and Self-Efficacy among Promotores de Salud?
$\underline{2016\ (n=61)}$ Undergraduate students Basic Sciences: 2 Clinical Sciences: 1 Population Sciences/Behavioral Sciences/Social Sciences: 18 Environmental Sciences: 7 Graduate/professional students Basic Sciences: 1 Clinical Sciences: 3 Population Sciences/Behavioral Sciences/ Social Sciences: 28 Environmental Sciences: 1 Undergraduate student poster winners 1st place: Chiamaka Ani, University of Georgia, Poster Title: The Role of Stat6 in Altering the Microbiota During Helminth Infection 2nd place: Joseph Real, The College of William & Mary, Poster Title: Stretched Thin: Social and Environmental Barriers to Diabetes Management among Low-Income Patients at a Safety Net Clinic 3rd place: Author Yao, California State University- Long Beach, Poster Title: Does “Confidence” Mediate the Relationship between English Proficiency and Health Status among Asian Americans? Graduate/professional student poster winners 1st place: Courtney Maclin-Akinyemi, University of Memphis, Poster Title: Profiles of African American Identity, Activity Engagement, SES, and Comorbid Obesity-Hypertension: Implications for Culturally Tailored Intervention Development 2nd place: Angelina Majeno, California State University- Long Beach, Poster Title: Differences in Mental Health and Well-Being among Women Living in Long Beach, California 3rd place: Christina Gladney, University of Florida, Poster Title: The Effects of Father Absence on Daughter’s Development	$\underline{2017\ (n=36)}$ Undergraduate students Basic Sciences: 3 Clinical Sciences: 0 Population Sciences/Behavioral Sciences/Social Sciences: 12 Environmental Sciences: 1 Graduate/professional students Basic Sciences: 1 Clinical Sciences: 1 Population Sciences/Behavioral Sciences/Social Sciences: 18 Environmental Sciences: 0 Undergraduate student poster winners 1st place: Abbas Berjaoui, Wayne State University, Poster Title: Health Disparities among Arab Americans in Michigan: Results from the 2013 Arab Behavioral Risk Factors Survey 2nd place: Mariam Khan, Santa Clara University, Poster Title: Understanding the Effects of Microaggressions and Acculturation Stress on the Mental Health of College Students 3rd place: Melody Nasser, California State University- Monterey Bay, Poster Title: Evaluating Pathways to Homelessness in LGBTQ-Identifying Residents of San Francisco Graduate/professional student poster winners 1st place: Mahalia Sam- Clarke, Claflin University, Poster Title: Directed Evolution of Temperature Dependence of Activity in α/β Barrel Enzyme 2nd place: Janelle K. Dunne-Wylie, George Washington University, Poster Title: Patient Experiences with Family Planning in FQHCs: Disparities by Race, Ethnicity and Language 3rd place: Monique McLeary, University of North Carolina- Greensboro, Poster Title: Support of Disordered Eating Behaviors in Online Spaces: A Content Analysis of the ProED Subreddit
$\underline{2018\ (n=21)}$ Undergraduate students Basic Sciences: 0 Clinical Sciences: 1 Population Sciences/Behavioral Sciences/Social Sciences: 3 Environmental Sciences: 1 Graduate/professional students Basic Sciences: 0 Clinical Sciences: 0 Population Sciences/Behavioral Sciences/Social Sciences: 16 Environmental Sciences: 0 Undergraduate student poster winners 1st place: David Chime, Allen University, Poster Title: The Effects of Crude Oil Spillage and Eventual Bioaccumulation in Humans 2nd place: Victoria Rai, University of Michigan, Poster Title: Unmet Need for Fetal Assessment amongst High-Risk Pregnancies in a Rural District Hospital 3rd place: Monica Taneja, John Hopkins University, Poster Title: Lifestyle, Ethnicity, and Inflammation Prevalence among Breast Cancer Survivors and Women Without Breast Cancer: A Comparison Using NHANES Graduate/professional student poster winners 1st place: Donica’ Beckett, University of Nevada- Los Vegas, Poster Title: Exploring PrEP Attitudes, Barriers and Facilitators of Use, Sexual Risk Behaviors and Communication Channel Preferences of Self-Reported Heterosexual African American/Black Students Enrolled in Jefferson County, Texas Colleges 2nd place: Elizabeth Duxbury, University of California- San Diego, Poster Title: Associations Between Education, Physical and Emotional Health, and Social Support in San Diego 3rd place: Veronica Morawek, The Catholic University of America, Poster Title: Psychosocial Factors Associated with Health Perception in Chronic Kidney Disease Patients. Alejandra Kaplan, Montclair State University, Poster Title: Exploring Acculturation as it Relates to Sociodemographic and Behavioral Factors Underlying Racial/Ethnic Disparities in Cancer Prevention Behaviors in New Jersey	

^*^The NCHD SRF was not held in 2015

## Discussion

Since 2011, the NCHD SRF Planning Committee has led the coordination of the annual SRF of the NCHD. The all-day NCHD SRF for undergraduate and graduate/professional students includes a poster session, oral presentations, a luncheon keynote speaker, and roundtable discussions. The NCHS SRF also includes an interactive learning module presented by a National Library of Medicine staff member.

The NCHD SRF appears to be reaching its focal group of participating students, African Americans and Hispanics/Latinx, who are the most underrepresented groups in the biomedical sciences. These two groups had the highest representation among all of the students who participated in the NCHD SRF to date.

From 2011 to 2016, academic partners in the cities where the NCHD SRF took place were identified and assisted with the recruitment of students. However, in 2017 and 2018, academic partners were not identified who could serve in this capacity. The lack of a local academic partner likely contributed to the lower numbers of participating students in 2017 and 2018 as compared with previous years. In the future, the NCHD SRF Planning Committee could enhance their efforts to include academic partners in several states with large numbers of Hispanic/Latinx students, including Arizona, New Mexico, Missouri, and Colorado.

The quality of the poster and oral presentations has improved steadily over the years as a result of the mandatory pre-conference training webinars. The NCHD SRF has included students from many regions of the US, including the Northwest, Southwest, Northeast, and Southeast. However, in the future, greater efforts could be made to advertise the NCHD SRF in Midwestern states such as Montana, North Dakota, South Dakota, Wyoming, Colorado, Utah, and Arizona. Some of these states have large Native American and Hispanic/Latinx populations, and research in these groups would be ideally suited for presentation during the NCHD SRF.

Given the increasing interest and success of the program, the NCHD SRF has since expanded to also offer mentorship and guidance related to academic and professional career development. For example, this has included roundtable discussions on applying to graduate/professional school for undergraduate students. For graduate/professional students, funding, mentoring, and making ethical decisions have been topics of discussion. Prior to the next NCHD SRF, its leaders will add a Q&A section to the NCHD SRF website to address past student’s questions to assist others in the future as well as continuing to respond individually to each student’s questions.

While the NCHD SRF has proven to be a successful research training opportunity for undergraduate and graduate/professional students, its scope could be expanded to include high school students, who are at earlier stages of the research training pipeline. To accomplish this goal, in the future, the SRF Planning Committee could reach out to local high school administrators to invite their students to participate in the SRF.

The NCHD SRF Planning Committee could also invite high school, undergraduate, and graduate/professional students to join the Committee to provide input on the overall topics as well as the activities that take place during the NCHD SRF. Additionally, the NCHD SRF Planning Committee could develop specific training activities related the education and career development of the high school students.

### Impact of the NCHD SRF

The NCHD SRF has had significant impacts on the career trajectories of many of the student participants. For example, a previous Hispanic/Latinx NCHD SRF poster presenter was a doctoral student at MUSC. The work that she presented at the NCHD SRF was based on a Diversity Supplement. She is now an Epidemiologist at a health research center in Florida.

Another NCHD SRF participant presented a poster while she was a doctoral student at the University of Nevada Las Vegas. She is Native American and is now an Assistant Professor in a College of Population Health. She recently identified five of her underrepresented Master of Public Health (MPH) students and helped them to submit abstracts of their scientific work to the 2020 NCHD SRF (which was postponed due to the COVID-19 pandemic).

A previous NCHD SRF participant later became the Director of Health Equity for the Health Commission of a major urban area. Her role in this position is to promote health equity in the city’s health and health care practices. She stood during an NCHD and publicly stated that her participation in the NCHD SRF solidified her commitment to a career in health equity and gave her the confidence to pursue her current role.

### Sustainability

From 2011 to 2014, the NCHD covered all of the travel costs of the student participants, including air travel, hotel costs, and meals. After 2014, the full travel scholarships grew too cost-prohibitive for the NCHD. Therefore, starting in 2016, the NCHD has supported the hotel costs of 20 students (with two students per hotel room), and supported the full travel costs of the two oral presenters (one undergraduate student and one graduate/professional student). The NCHD SRF also formed a Financial Assistance Sub-Committee consisting of three individuals who contact the research mentors, faculty advisors, chairs, and deans of the students whose abstracts were accepted to help them to identify potential sources of funding at each student’s academic institutions, as well as potential funding from other sources such as fraternities and sororities, local businesses, and scientific organizations.

Funding is needed to sustain the NCHD SRF and attendance at the annual meeting which takes place at a different institution each year. Local and national partners are actively being identified and welcomed to build relationships with individuals and organizations committed to improving health and development.

We accomplish this through various levels of sponsorship opportunities ($5,000; $10,000; $25,000+; and $50,000+) and benefits such as complimentary conference registrations and exhibit space, logo placement and URL link on the conference website, and inclusion in the Souvenir Conference Program, among others.

## Strengths and Limitations

While the change in funding support for the NCHD SRF appears to have resulted in a smaller number of students who participate each year, it has also resulted in a broader geographic distribution of the academic institutions of the participating students. A limitation of the NCHD SRF is the higher rate of conference participation among women when compared with men. In the future, the NCHD SRF organizers will make an effort to increase the participation of men by reaching out to local community groups and student organizations including barbershops, fraternities, social justice organizations, science clubs, Reserve Officer Training Corps (ROTC) groups, student unions, athletic clubs and sports leagues, and churches.

## Conclusion

The NCHD SRF differs from many existing national meetings in that it provides distinct value-added activities including capacity-building, research training, and presentation experience that are not available in other national meetings that are valuable to both undergraduate and graduate/professional students alike. Additionally, the NCHD SRF focuses on underrepresented groups in STEM, particularly African Americans/blacks and Hispanic/Latinx students. These include guidance in abstract development, a webinar on presentation techniques and methods, a vibrant student-centric conference, and professional development workshops on finding a mentor and locating scholarship/fellowship funding, networking, and strategies for handling ethical issues in research with mentors. In summary, the NCHD SRF presents an ideal opportunity to invest in future leaders through career and professional development activities, including mentorship.

## Supplementary Information

Below is the link to the electronic supplementary material.

Supplementary file1 (DOCX 16 KB)

## Data Availability

NCHD SRF data are published annually in a report and available online.

## Code availability

SPSS (IBM Corp. Released 2017. IBM SPSS Statistics for Windows, Version 25.0. Armonk, NY: IBM Corp.) was used for data analysis and is available upon request.
