# Supplementary material for: The National Conference on Health Disparities Student Research Forum
Source: J Cancer Educ. 2021 Oct 16;38(1):85–95. doi: 10.1007/s13187-021-02082-3 (PMC8519742; doi:10.1007/s13187-021-02082-3)
Supplement: Supplementary file 1 — Supplementary file1 (DOCX 16 KB) [file 13187_2021_2082_MOESM1_ESM.docx]

**Supplemental Table. Locations of the National Conference on Health Disparities (NCHD) and the Academic Institutions of the Student Research Forum (SRF) Participants, 2011-2018^a^ in Alphabetical Order**

| **NCHD 2011**  **Charleston, SC** | **NCHD 2012**  **Little Rock, AR** | **NCHD 2013**  **St. Thomas, USVI** | **NCHD 2014**  **Long Beach, CA** | **NCHD 2016**  **Washington, DC** | **NCHD 2017**  **New Orleans, LA** | **NCHD 2018 Philadelphia, PA** |
| --- | --- | --- | --- | --- | --- | --- |
| Allen University in South Carolina | Allen University in South Carolina | California State University Long Beach | Allen University in South Carolina | Allen University in South Carolina | Allen University in South Carolina | Allen University in South Carolina |
| California State University, Long Beach | Arkansas Baptist College | Clemson University in South Carolina | California State University - Dominquez Hills | California State University Long Beach | California State University - Monterey Bay | Baylor College of Medicine in Texas |
| Claflin University in South Carolina | Benedict College in South Carolina | Emory University in Atlanta | California State University - Fullerton | California State University Monterey Bay | California State University -Northridge | Boston College in Massachusetts |
| College of Charleston in South Carolina | California State University Long Beach | Georgia Southern University | California State University - Long Beach | Charles Drew University in California | Case Western Reserve University in Ohio | California State University - Monterey Bay |
| Medical University of South Carolina | College of Charleston in South Carolina | Jackson State University in Mississippi | California State University - Monterey Bay | College of William & Mary in Virginia | Claflin University in South Carolina | The Catholic University of America in Washington, DC |
| South Carolina State University | Georgia Southern University | Johns Hopkins University in Maryland | California State University - Northridge | Columbia University in New York | Elon University in North Carolina | College of Charleston in South Carolina |
|  | Georgia State University | Medical University of South Carolina | California State University - San Bernardino | Drexel University in Pennsylvania | George Washington University in Washington, DC | Drexel University in Pennsylvania |
|  | Johns Hopkins University in Maryland | Morehouse School of Medicine in Georgia | Columbia University in New York | East Tennessee State University | Quinnipiac University in Connecticut | Georgia Southern University |
|  | Harvard School of Public Health in Massachusetts | Morgan State University in Maryland | Georgia State University | Eastern Virginia Medical School | Santa Clara University in California | Johns Hopkins University in Maryland |
|  | Medical University of South Carolina | Rensselaer Polytechnic Institute in New York | Medical University of South Carolina | Emory University in Georgia | Tulane University in Louisiana | Long Beach Memorial Medical Center in California - Family Medicine |
|  | Morehouse College in Georgia | Rowan University in New Jersey | Meharry Medical College in Tennessee | Goethe University Frankfurt Germany | University of Florida | Medical University of South Carolina |
|  | Philander Smith College in Arkansas | Spelman College in Georgia | Morehouse School of Medicine | Manhattan College in New York | University of Illinois - Chicago | Montclair State University in New Jersey |
|  | Spelman College in Georgia | University of Arkansas for Medical Sciences | Occidental College in California | Medical University of South Carolina | University of Miami Miller in Florida | Ohio State University |
|  | University of Arkansas for Medical Sciences | University of Nevada - Las Vegas | San Diego State University | Rochester Institute of Technology in New York | University of North Carolina -Greensboro | Rowan University in New Jersey |
|  | University of Bedfordshire, UK | University of South Carolina | Spelman College in Georgia | Spelman College in Georgia | University of Southern California | State University of New York - Albany |
|  | University of Florida | University of Toledo Medical Center in Ohio | Tufts University in Massachusetts | State University of New York Downstate | University of Texas -El Paso | Tuskegee University in Alabama |
|  | University of Michigan | University of the Virgin Islands | University of California -Davis | Texas A & M University | University of Virginia | University of Arkansas |
|  | University of North Carolina Chapel Hill | University of the West Indies - Mona | University of California - Irvine | Texas Tech University | University of Washington | University of California - San Diego |
|  | University of South Carolina | University of Wisconsin | University of Illinois- Chicago | University of California - Irvine | University of Wisconsin-Madison | University of Memphis in Tennessee |
|  | Vanderbilt School of Medicine in Tennessee |  | University of Kansas | University of Colorado - Denver | Walden University | University of Michigan |
|  |  |  | University of Massachusetts | University of Florida | Xavier University of Louisiana | University of Nevada - Las Vegas |
|  |  |  | University of North Carolina - Chapel Hill | University of Georgia |  | University of North Texas Health Science Center at Fort Worth |
|  |  |  | University of Southern California | University of Kansas |  | University of South Carolina |
|  |  |  | University of Texas - Austin | University of Kentucky |  | University of Texas Health San Antonio |
|  |  |  | University of the Virgin Islands | University of Memphis in Tennessee |  | University of Washington |
|  |  |  | Wayne State University in Michigan | University of Michigan - Ann Arbor |  | University of Wisconsin - Madison |
|  |  |  |  | University of North Carolina - Chapel Hill |  |  |
|  |  |  |  | University of Southern California |  |  |
|  |  |  |  | University of the Virgin Islands |  |  |
|  |  |  |  | University of Wisconsin - Madison |  |  |
|  |  |  |  | University of Wisconsin - Green Bay |  |  |
|  |  |  |  | Virginia Commonwealth University |  |  |

Abbreviations: SC, South Carolina; AR, Arkansas; UK, United Kingdom; USVI, United States Virgin Islands; CA, California; DC, District of Columbia; LA, Louisiana; PA, Pennsylvania.

^a^ The NCHD SRF was not held in 2015.
